# What Is Wellbeing?

**DOI:** 10.3390/ijerph20065006

**Published:** 2023-03-12

**Authors:** Aaron Jarden, Annalise Roache

**Affiliations:** 1 Centre for Wellbeing Science, Melbourne Graduate School of Education, University of Melbourne, Melbourne 3010, Australia; 2 Psychology, Health Sciences, Auckland University of Technology, Auckland 0627, New Zealand

The human interest in ‘wellbeing’ dates back thousands of years, with complex indigenous understandings [1] through to the ancient Greeks [2]. The notion of wellbeing has endured the test of time and is contemporarily central to the social sciences. As Alexandrova [3] highlights, “concern with human wellbeing is at the very root of modern social science social science thus began its life as a form of knowledge devoted officially to the advancement of wellbeing” [4] (pp. 3–4). Particularly interesting, however, is that ‘wellbeing’ also constitutes an emergent hot topic across the social sciences, with disciplines ranging from psychology and economics to sociology, philosophy, literature, developmental studies, health, communication and media, education, public policy and beyond, all placing greater emphasis on the term and construct than previously. It is clear that the rapid and phenomenal growth of the field of positive psychology [5,6,7] has contributed to stimulating interest in wellbeing more broadly [8]. Today, many scholars across most social science discipline, at least from a publishing perspective, are taking a wellbeing-oriented lens to their field and discipline. Additionally, beyond academia and in the realm of popular culture (at least in western societies), wellbeing is becoming more central in various dialogues, with the acknowledgment that wellbeing (in contrast to illbeing) is important for everyone. For example, Seligman notes that when parents are asked what they most want for their children, wellbeing is usually present in their answers and also usually at the top of the list [9].

However, a clear and useful definition and conceptualisation of wellbeing remains elusive, a situation which has manifested historically, contemporarily, to the public, and within and across disciplines. As Hone and colleagues mentioned, “although several researchers and research teams have developed theoretical, conceptual and operational models of wellbeing, and there is general agreement that wellbeing is a multi-dimensional concept, that is where the consensus ends” [10]. Disciplines define wellbeing in a variety of different ways, if they any definition is given at all. Some definitions are coherent yet board, and arguably vague and imprecise, such as “how well someone’s life is going for them” [11]. Conversely, others are less clear and have multiple different components, such as “a state of happiness and contentment, with low levels of distress, overall good physical and mental health and outlook, or good quality of life” [12]. One of the most widely cited definitions of wellbeing is as follows: “wellbeing can be understood as how people feel and how they function both on a personal and social level, and how they evaluate their lives as a whole” [13] (p. 6). This characterisation points to wellbeing having multiple elements within and across the broad categories of emotion, behaviour, cognition, and relationships. Nonetheless, definitions such as these seem a long way from the way ordinaary people use the word ‘wellbeing’. If the philosopher Wittgenstein is correct in that “the meaning of a word is its use in the language” [14] (Section 43), then there is potentially a large discrepancy gap between the words meaning and its use. This would be hugely problematic for any progress or communication.

Additionally, some of the research to date points to vast differences in how laypeople, in contrast to academics, conceptualise wellbeing. For example, central to a concept of wellbeing for laypeople are the values mental health, feeling valued, work–life balance, and the notion of inner harmony. Conversely, these are not present in academic models, which usually focus on components such as meaning and relationships [10,15,16,17]. In addition, differences across the lifespan have also been identified. For example, adolescents report that happiness, kindness, fun and safety are central to their conceptualisation of wellbeing. In contrast, for older individuals, these are more peripheral or not important, and components such as meaning and purpose are not important for adolescents, whereas they are for older individuals [18]. Cultural differences are also apparent in wellbeing’s conceptualisations. For example, Huang and colleagues found that optimism and contentment were central to wellbeing for Chinese students, whereas components that Europeans place as centrally important, such as mental health, were not so important [10]. Furthermore, there is a growing dilution of the term through corporate initiatives which appear only superficial in their commitment to employee wellbeing, termed ‘wellbeing washing’, a process which is further compounded by the commercial appropriation and monetisation of the term in the form of ‘wellbeing’-related products.

For these reasons, we can observe the conceptualisation and clear definition of wellbeing as an essential first step in the successful development and implementation of any wellbeing theory, policy, activity, debate, or intervention and as being at the base of any scientific attempt to measure or improve wellbeing. As such, a comprehensive and socially relevant research approach needs to be established to understand better what wellbeing is and what it means to people. In addition to the clarity of ‘wellbeing’, clarity is also required on similar terms and terms that wellbeing is highly related to. As such, we suggest similar challenges are present for terms such as: wellness; flourishing; thriving; life satisfaction; happiness; quality of life; mental health; and subjective wellbeing, to name only a few.

The state of play seems to be that, despite wellbeing becoming an increasingly popular term and topic, many disciplines do not define wellbeing. When they do, they do not define wellbeing satisfactorily or clearly, and across disciplines, definitions and conceptualisations vary markedly. This is also the case in the public discourse, especially with different media and mediums, as similar terms are used interchangeably. The research literature suggests significant differences in conceptualisations of wellbeing across different cultures, ages, and population groups (e.g., nurses vs. teachers). Academics agree that wellbeing is a multi-dimensional concept, but disagree as to what those dimensions and components are. Such a state of play is problematic because:

(1) Large literature bases, such as that in the field of positive psychology, rest on what is meant by ‘wellbeing’;
(2) It makes talking to and working with different academic disciplines challenging and limits interdisciplinary research;
(3) It presents a conundrum for practitioners when it comes to more practical aspects, such as wellbeing model selection or the measurement of wellbeing, and
(4) It leaves the term prone to misuse and misapplication, which may weaken its utility as a concept.

Therefore, this Special Issue was devised as a forum for researchers to contribute to and thus strengthen the field. In particular, we have called for submissions that assist in the process of understanding what wellbeing is, or that use new ways or approaches to shine a light on the notion of wellbeing or how we might understand it better. To date, we have accepted five articles that help with this mission, and in the following section, we highlight their contributions.

Firstly, Lomas and VanderWeele argue that our understanding of wellbeing and related concepts such as health and flourishing is shaped by the metaphors through which we think about such ideas. Although we currently think about wellbeing through metaphors such as a pyramid, ladder, and continuum, they provide two new metaphors of a garden and of an orchestra which they claim better do justice to the nuanced complexities of wellbeing. Secondly, Cebral-Loureda, Tamés-Muñoz, and Hernández-Baqueiro provide a bibliometric review of the concept of human flourishing, which is sometime being referred to as having high levels of wellbeing. Here, statistical methods and data mining were used to analyse thousands of documents related to the term “human flourishing”. Through cluster and network analyses, the authors show the concept’s evolution, composition, and current tensions and trends. Thirdly, Rusk proposes a new model which provides insight into the ‘how’ and ‘why’ of wellbeing to understand better the ‘what’ of wellbeing (or what wellbeing is). Informed by evolutionary psychology and neuroscience, the model proposes that systems for adaptive motivation underpin experiential and reflective wellbeing. The proposed model differentiates four layers of wellbeing: objective, experiential, reflective, and narrative, which relate to the model in different ways. The model integrates the constituents of wellbeing, human motives, and specific emotions, suggesting that wellbeing arises directly from a system for adaptive motivation and offers a set of fundamental principles and processes that may underlie diverse conceptualisations of wellbeing. Lastly, Joshanloo offers two articles that provide insight into wellbeing. The first focuses on mental balance, defined as a sense of tranquillity resulting from inner peace and harmonious interactions with the external environment, and argues that this is a necessary but largely overlooked aspect of wellbeing. The second focuses on cross-group differences in life satisfaction from a measurement perspective and elaborates whether this should be understood similarly or not. Here, the results suggest that across groups, such as those based on gender, age, ethnicity, national background, etc., all groups understand and answer questions about life satisfaction similarly, meaning that life satisfaction can be meaningfully compared between groups. Such a finding shows the promise that wellbeing can likewise be investigated and compared between similar groups from a psychometric perspective.
